# Advanced Physical-Layer Technologies for Beyond 5G Wireless Communication Networks

**DOI:** 10.3390/s21093197

**Published:** 2021-05-04

**Authors:** Waqas Khalid, Heejung Yu, Rashid Ali, Rehmat Ullah

**Affiliations:** 1 Institute of Industrial Technology, Korea University, Sejong 30019, Korea; waqas283@korea.ac.kr; 2 Department of Electronics and Information Engineering, Korea University, Sejong 30019, Korea; 3 School of Intelligent Mechatronics Engineering, Sejong University, Seoul 05006, Korea; rashidali@sejong.ac.kr; 4 School of Electronics, Electrical Engineering and Computer Science, Queen’s University, Belfast BT9 5BN, UK; r.ullah@qub.ac.uk

**Keywords:** B5G, 6G, physical-layer technologies

## Abstract

Fifth-generation (5G) networks will not satisfy the requirements of the latency, bandwidth, and traffic density in 2030 and beyond, and next-generation wireless communication networks with revolutionary enabling technologies will be required. Beyond 5G (B5G)/sixth-generation (6G) networks will achieve superior performance by providing advanced functions such as ultralow latency, ultrahigh reliability, global coverage, massive connectivity, and better intelligence and security levels. Important aspects of B5G/6G networks require the modification and exploitation of promising physical-layer technologies. This Special Issue (SI) presents research efforts to identify and discuss the novel techniques, technical challenges, and promising solution methods of physical-layer technologies with a vision of potential involvement in the B5G/6G era. In particular, this SI presents innovations and concepts, including nonorthogonal multiple access, massive multiple-input multiple-output (MIMO), energy harvesting, hybrid satellite terrestrial relays, Internet of Things-based home automation, millimeter-wave bands, device-to-device communication, and artificial-intelligence or machine-learning techniques. Further, this SI covers the proposed solutions, including MIMO antenna design, modulation detection, interference management, hybrid precoding, and statistical beamforming along with their performance improvements in terms of performance metrics, including bit error rate, outage probability, ergodic sum rate, spectrum efficiency, and energy efficiency.

## 1. Introduction

Fifth-generation (5G) networks support various service categories, including enhanced mobile broadband (eMBB), ultrareliable low-latency communications (URLLC), and massive machine-type communications (mMTC), and enable advanced functions such as lower latency, greater capacity, and optimized support for Internet of Things (IoT) networks [1]. The key enabling technologies of 5G networks include multiple-input multiple-output (MIMO), nonorthogonal multiple access (NOMA), energy harvesting, and millimeter-wave (mmWave) communications [2]. The deployment and standardization activities of 5G networks are rapidly increasing. The 3GPP Release 17 (Rel-17) marks an important chapter in the standardization of 5G new radio (NR). Target features in Rel-17 include massive MIMO, support for the 52.6–71 GHz spectrum band, support for multicast/broadcast, and enhancements in terms of integrated access and backhaul, industrial IoT/URLLC, 5G NR sidelink, and dynamic spectrum sharing. Despite these efforts, 5G is in its early stages, and many operators are yet to announce launches of commercial 5G services [3]. The conceptualization of beyond 5G (B5G)/sixth-generation (6G) networks has begun in parallel to sustain the competitive edge of wireless communication networks. The global number of Internet-connected devices is expected to exceed 50 billion, and the volume of wireless data traffic providing emerging IoT services is predicted to be 5016 exabytes/month [4,5]. The B5G/6G networks with technological advantages and demanding disruptive capabilities are expected to provide solutions to the ubiquitous connectivity and spectral demands in 2030 and beyond.

The 6G networks would extend the 5G application scenarios (i.e., further-eMBB, ultra-mMTC, enhanced-uRLLC) and introduce new application scenarios, including ultrahigh-density data, ultrahigh-speed low-latency communications, and ubiquitous mobile ultrabroadband. Compared to 5G networks, 6G networks could achieve superior performance and offer new performance metrics. The key features of 6G networks are an uplink data rate of up to 1 Tbps (compared to 10 Gbps in 5G), spectral efficiency of up to 1000 bps/Hz/m${}^{2}$ (compared to 10 bps/Hz/m${}^{2}$ in 5G), reliability up to 10${}^{-9}$ frame error rate (compared to 10${}^{-5}$ frame error rate in 5G), mobility support of up to 1000 km/h (compared to 500 km/h in 5G), control-plane latency of less than 1 ms (compared to 10 ms in 5G), processing delay of 10 ns (compared to 100 ns in 5G), traffic capacity of up to 1–10 Gbps/m${}^{2}$ (compared to 10 Mbps/m${}^{2}$ in 5G), localization precision of 1 cm on 3D (compared to 10 cm on 2D in 5G), uniform user experience of up to 10 Gbps 3D (compared to 50 Mbps 2D in 5G), and full integration of artificial intelligence (AI) [6]. The potential evolutionary services/technologies of 6G networks include joint sensing and communication, reconfigurable intelligent surfaces (RISs), holographic MIMO surfaces and beamforming, free duplexing and spectrum sharing, massive device-to-device (D2D) communication, nonterrestrial communication, E-health and biosensing, automated vehicles and robotics for Industry 4.0 and beyond, tactile Internet, dynamic network slicing, and software-defined networking and network-function virtualization. In addition, the potential of radio signaling and maximal cognitive for intelligent radio transmission can be fully realized through AI-empowered systems with the aid of machine-learning (ML) algorithms. Simultaneously, the potential major challenges of 6G networks include communications in higher bands, device capabilities, network security, and transceiver and antenna designs [7,8].

Abbreviations used in this manuscript are listed in Table 1.

The rest of the editorial is organized as follows. Section 2 provides the overview of the Special Issue. In Section 3, we present a brief review of the technical papers. Lastly, Section 4 concludes the editorial.

## 2. Special Issue Overview

The motivation behind this Special Issue (SI) of MDPI SENSORS was to consolidate all the stakeholders, and the entirety of academia and industry, and solicit the cutting-edge original research to identify and discuss the latest concepts, innovations, standards, potential use cases, open research problems, technical challenges, and promising solution methods in B5G/6G systems from the perspective of the physical layer. Accepted papers in this SI covered a wide range of research in the broader domain of physical-layer technologies to be implemented in the B5G/6G era. Topics of interest for this SI include:

- Massive MIMO systems, such as advanced massive MIMO, cell-free massive MIMO, and beamspace massive MIMO.
- Adaptive signal processing, and analytical modeling and design.
- Novel modulation and coding techniques.
- Radio resource and interference management.
- Communications over terahertz (i.e., 0.1–10 THz), and mmWave (i.e., 30–300 GHz) bands.
- Emerging networks such as wirelessly powered, aerial-access (e.g., unmanned aerial vehicles, satellites, etc.), vehicular ad hoc, and cooperative relaying networks.
- Advanced solutions, such as NOMA, RISs, joint sensing, and communication.
- Implementation and challenges of physical-layer security.
- Licensed or unlicensed spectrum interoperability and dynamic spectrum allocation.
- Requirements and solutions for the 5G NR/6G physical layer.
- Energy-efficient operations (e.g., energy harvesting, wireless power transfer).
- Internet of Everything.
- Mobile edge/fog computing.
- AI techniques and ML/deep-learning algorithms for physical-layer design and optimization, including MIMO signal detection, real-time channel estimation, multiuser detection, and channel modeling and propagation.

The theme of the SI is reflected in the accepted papers; 25 papers were submitted, out of which only the top 15 research articles were accepted and published.

## 3. Brief Review of Technical Papers

NOMA and cognitive-radio (CR) networks were envisioned as key enabling techniques for 5G networks. The primary approach in NOMA is to remove orthogonality between allocated resource blocks, and it enables the simultaneous transmission of multiple users. Consequently, such an approach improves transmission rate and user fairness in the system, and provides superior spectrum utilization efficiency. Similarly, flexible spectrum allocation and efficient spectrum utilization can be achieved using CR technology [9,10]. In the first paper, titled Performance Optimization of Hybrid Satellite-Terrestrial Relay Network Based on CR-NOMA [11], the authors proposed a joint relay-and-antenna selection scheme on the basis of the NOMA–CR scenario to achieve the maximal communication rate of the secondary user under a condition in which the primary user maintained optimal outage performance. Simulation results proved the effectiveness and superiority of the proposed scheme, and indicated the impact of parameter configurations on system outage performance.

The integration of MIMO and relaying systems is a promising solution for the reliability and coverage improvements in 5G networks. However, the benefits of these technologies can only be achieved using proper system design. In the second paper, titled Separable MSE-Based Design of Two-Way Multiple-Relay Cooperative MIMO 5G Networks [12], the authors considered an interesting problem of jointly optimizing terminal precoders/decoders and relay forwarding matrices on the basis of the sum mean square error (MSE) criterion in MIMO two-way relay systems. Rather than attempting to iteratively solve the optimization problem, a relaxed version of the original minimal sum MSE nonconvex optimization problem was derived, which enabled the construction of two simpler problems that admitted the closed-form, albeit suboptimal, solution. The proposed technique exhibited performance gain over the previous iterative method proposed in [13].

The development of hybrid satellite-terrestrial relay networks (HSTRNs) is a driving force in revolutionizing satellite communications in the modern era. The use of HSTRNs is required for the seamless integration of terrestrial cellular and satellite communications. Moreover, more users are simultaneously served using NOMA, and different performance types or services are observed among these users [14]. In the third paper, titled Hybrid Satellite-Terrestrial Relay Network: Proposed Model and Application of Power Splitting Multiple Access [15], the authors considered the requirements of both performance and energy efficiency (EE) for the future satellite communications, and provided an indepth performance evaluation of NOMA-enabled HSTRNs with a wireless-powered (i.e., energy harvesting) terrestrial relay. The proposed analytical expressions and derived results provided design insights for the implementations of HSTRNs in the 5G/6G era.

Spectral efficiency (SE) is a highlighted feature of 5G/B5G MIMO systems. In the fourth paper, titled Spectral-Efficiency Augmentation in Uplink Massive MIMO Systems by Increasing Transmit Power and Uniform Linear Array Gain [16], the authors examined a massive MIMO system to increase the SE in each cell and consequently the area throughput of the proposed system. The presented analytical modeling indicated the possibility of maximizing area throughput by determining appropriate parameters in terms of average cell density, available bandwidth, and SE. The effectiveness of the proposed model was validated using simulation results implemented in real-time scenarios. The findings of the paper are useful in ensuring the efficient transmissions in future massive MIMO systems.

Hybrid precoding strategies provide a potential solution to the path loss experienced by mmWave-MIMO systems. Furthermore, improved spectrum utilization is a key requirement in 5G/B5G systems. Consequently, the design of spectrally efficient precoders for partially connected hybrid structures is important. Evolutionary algorithms for the joint computation of RF/digital precoders increase the SE of a partially connected hybrid precoding architecture. The paper titled Evolutionary-Algorithm-Based Capacity Maximization of 5G/B5G Hybrid Precoding Systems [17] considered the problem of spectrally efficient precoder design and presented an evolutionary-algorithm-based hybrid precoding strategy for partially connected antenna arrays in mmWave-massive MIMO systems. The proposed artificial bee-colony-based precoding scheme provided the highest achievable rates and outperformed benchmark evolutionary algorithms in terms of SE.

The 5G IoT-enabled smart-home environment detects human activities by manipulating data collected from numerous sensors and using appropriate deep-learning algorithms. The detection of expected or unexpected behavior events indicating human activity is a challenging task in the activity-detection paradigm. In addition, sensor energy resources are continuously depleted for the ubiquitous and extensive monitoring of activities. The paper titled Scheduling Sensor Duty Cycling Based on Event Detection Using Bidirectional Long Short-Term Memory and Reinforcement Learning [18] presented an energy- and event-aware sensor duty cycling (EEA-SDC) scheme. In the proposed scheme, the event of a resident’s activities in a smart home was modeled as a time-sequence information problem, and the bidirectional long short-term memory (LSTM) model was used to predict expected future events and allocate the predictive sensors. Moreover, a monitor sensor within a cluster of hibernate sensors using the Jaccard similarity index was used to detect the unexpected events. The performance of the proposed scheme was optimized by employing a Q-learning algorithm by tracking missed or undetected events.

Different users in multiuser 5G MIMO networks can experience distinct spatial channel correlations. The paper titled Statistical Beamforming for Massive MIMO Systems with Distinct Spatial Correlations [19] presented a novel statistical beamforming (SBF) method called partial-nulling-based SBF (PN-SBF) to serve several users experiencing distinct spatial channel correlations. The massive MIMO system with the two user groups was considered, i.e., the first group experienced low spatial channel correlation, and the second group experienced high spatial channel correlation. A prebeamforming matrix based on the zero-forcing-based design principle was constructed to eliminate intergroup interference from the low- to the high-correlation group. Furthermore, postbeamforming vectors were designed to maximize the signal-to-leakage-and-noise ratio to solve intragroup interference. Simulation results indicated that the proposed PN-SBF scheme performed better than conventional SBF schemes did in terms of the ergodic sum rate in a massive MIMO system with distinct spatial correlations.

RF energy harvesting is a promising solution for EE in 5G systems [20]. In the paper titled Outage Probability and Ergodic Capacity of a Two-User NOMA Relaying System with an Energy Harvesting Full-Duplex Relay and Its Interference at the Near User [21], the authors considered the two-user downlink NOMA relay system in which a full-duplex and energy-harvesting-enabled relay assisted communications between the base station and a far user over flat, independent, and nonidentically Rayleigh fading channels. The paper provided insights into the impact of interuser interference at the relay and self-interference cancellation coefficient on the outage probabilities and ergodic capacities. Furthermore, the optimal value of the power-division ratio was determined. Numerical results indicated that the decoding signal-to-interference-plus-noise ratio decreased when the relay harvested more energy; consequently, outage-probability performance and ergodic capacity at the far user were reduced. Using the proposed numerical method, an appropriate power-division ratio satisfying the quality-of-service requirements at the far user was determined.

D2D communication is a promising relay strategy for 5G networks. In the paper titled Device-to-Device Aided Cooperative Relaying Scheme Exploiting Spatial Modulation: An Interference-Free Strategy [22], the authors proposed a novel interference-free dual-hop D2D-aided cooperative relaying strategy based on spatial modulation (termed D2D-CRS-SM), and demonstrated the interference-free information reception with multiple cellular and D2D users operating in the same frequency band. The symbol and antenna indices of a transmitter were the desired pieces of information of a single receiver in a conventional SM [23]. In contrast, the presented D2D-CRS-SM scheme considered symbol and antenna indices as the desired pieces of information of two different receivers; thus, it facilitated efficient multiuser communication. The performance of the proposed D2D-CRS-SM was studied in terms of bit error rate and SE considering M-ary phase-shift keying and quadrature amplitude modulation over independent Rayleigh fading channels. Lastly, the efficiency of D2D-CRS-SM was demonstrated via Monte Carlo simulations. Results demonstrated the usefulness of the proposed scheme in 5G cellular-D2D wireless communication networks.

The deployment of 5G technologies with home automation systems (HASs) has created a new ecosystem with significant potential. Challenges in HAS include the availability of infrastructure (e.g., Internet or electrical energy), human–appliance interactions, and the implementation of ML models in real time. To address the aforementioned automation challenges and control energy wastage owing to user lifestyles in smart homes, the paper titled Towards Energy Efficient Home Automation: A Deep-Learning Approach [24] proposed an autonomous smart-home system to automatically control energy consumption by employing machine- and deep-learning techniques. In detail, the proposed scheme was operated in three phases: (i) feature extraction and classification based on a 1-dimensional deep convolutional neural network, (ii) LSTM to forecast the load based on the extracted features in Phase 1, and (iii) a scheduling algorithm based on forecasted data to schedule the operational time of smart-home appliances. The proposed scheme efficiently automated the smart-home appliances with lower energy consumption while adapting to the lifestyle of the users. The validation of the proposed scheme was tested using several simulation scenarios incorporating datasets from authentic data sources. The simulation results indicated the advantage of the proposed HAS scheme in terms of fulfilling the energy demands of the users.

The increasing number of wirelessly connected devices in 5G IoT networks has increased the demand for MIMO technology to efficiently utilize the limited bandwidth. The large space at the base station enables the easy exploitation of MIMO technology. In contrast, complex procedures are required for mobile handsets because of limited space to increase the number of active antenna elements. The paper titled Dual Band and Dual Diversity Four-Element MIMO Dipole for 5G Handsets [25] proposed the design of a four-element MIMO dual-band, dual-diversity, dipole antenna for 5G-enabled handsets, and focused the space and pattern diversity to provide acceptable MIMO performance. The usefulness of the proposed 4×4 MIMO dipole antenna was verified by comparing simulated and measured results using a fabricated version of the proposed antenna. Specific absorption-rate analysis was performed using the CST Voxel model. The proposed antenna was tested to be operational in dual sub-6 GHz bands, as the envelope correlation coefficient, isolation, and capacity loss were demonstrated to be below the standard margin; thus, the antenna structure was appropriate for 5G MIMO applications.

The 5G mmWave communication enables the high-capacity delivery and better handling of peak data rates. The paper titled Interbeam Cochannel Downlink and Uplink Interference for 5G New Radio in mmWave Bands’ [26] presented a methodology of assessing cochannel interference for multibeam antennas in 5G MIMO systems. The developed methodology maximized the SE by using the same frequency bands for angularly separated antenna beams of a 5G base station (gNodeB). The presented multiellipsoidal propagation model (MPM) provided mapping of multipath propagation, and considered the directivity of antenna beams. Then, 5G microcell base stations operating in mmWave bands were analyzed. Similar approaches based on the 3GPP statistical model require the knowledge of the spatial parameters. In contrast, the proposed MPM approach considered signal-to-interference ratio assessments only; therefore, it was more suitable for any propagation scenarios. Results indicated that the proposed MPM approach provided faster determination of the same optimal directions for adjacent gNodeB beams.

MIMO is envisaged as a key enabling technology for 5G networks [27]. In the paper titled A Two-Hop mmWave MIMO NR-Relay Nodes to Enhance the Average System Throughput and BER in Outdoor-to-Indoor Environments [28], the authors presented the architecture of two-hop out-of-band relay nodes (RNs) with MIMO capabilities to enable 5G communications in the mmWave band. Two relaying strategies, i.e., amplify-and-forward and decode-and-forward, were implemented to assist communications in outdoor-to-indoor environments between 5G radio node and NR user equipment. The proposed scheme considered two different channel-estimation approaches: (i) the complete knowledge of a channel, (ii) and channel estimation through least-squares estimator. Furthermore, simulation results implemented in MATLAB and Simulink verified the effectiveness of the proposed two-hop mmWave MIMO NR-RNs architecture. Simulation results implemented with 5G features such as downlink shared-channel transport channel coding, synchronization-signal burst generation, and physical downlink shared-channel generation showed improvements for the proposed scheme in terms of bit error rate and throughput.

The design of modulation-detection techniques is critical in order to satisfy the diverse requirements of 5G services, as it determines the throughput, complexity, and reliability of the system from the perspective of the physical layer. The paper titled Machine Learning for 5G MIMO Modulation Detection [29] proposed a low-complexity modulation-detection algorithm for 5G multirelay cooperative MIMO systems over imperfectly estimated correlated channels. In detail, higher-order statistics of the received signal were extracted as discriminating features at the destination node. A comparative study between the random committee and AdaBoost ML techniques was established at a low SNR after applying principal-component analysis. The performance of the proposed modulation-detection scheme was analyzed in terms of various matrices, including recall, false-positive and true-positive rates, F measure, and time consumed to build the model. The superiority of the proposed scheme in terms of computational complexity and modulation detection was verified through simulation results.

The paper titled 6G-Enabled Smart Infrastructure for Sustainable Society: Opportunities, Challenges, and Research Roadmap [30] presented details on the evolution of wireless-communication standards from 1G to 5G, and insights into the panoramic vision of 6G networks. The comparative review with the limitations of related survey papers and contributions of the paper to fill the knowledge gap was extensively discussed. Then, potential enabling technologies and emerging applications of 6G networks were reviewed. In contrast to previous papers, this paper presented extensive analysis of the associated challenges with the actualization of 6G networks in terms of commercialization, social, and health, and new use cases in terms of education, transport, media, and logistics. Lastly, driving trends, open research problems, and probable solutions were discussed to present future research explorations in 6G networks.

## 4. Conclusions

In this SI, an overwhelming submission of 25 manuscripts were received in total, out of which only 15 high-quality papers were accepted for the publication after the rigorous peer review. This SI was initiated to consolidate researchers and industry professionals reporting on the recent research advances on physical-layer technologies to be implemented in the B5G/6G era. The aim was reflected in the accepted papers that presented the state-of-the-art research developments on this topic.

## Figures and Tables

**Table 1:** List of abbreviations.

Abbreviation	Description
5G	Fifth generation
eMBB	Enhanced mobile broadband
mMTC	Massive machine-type communications
uRLLC	Ultrareliable and low-latency communications
B5G	Beyond fifth generation
6G	Sixth generation
MIMO	Multiple-input multiple-output
mmWave	Millimeter wave
NOMA	Nonorthogonal multiple access
CR	Cognitive-radio
RISs	Reconfigurable intelligent surfaces
D2D	Device to device
IoT	Internet of Things
HAS	Home automation system
RN	Relay node
gNodeB	5G base station
SE	Spectral efficiency
EE	Energy efficiency
AI	Artificial intelligence

## Data Availability

Not applicable.

## References

[1] Yu H., Lee H., Jeon H. (2017). What is 5G? Emerging 5G Mobile Services and Network Requirements. Sustainability.

[2] Khalid W., Yu H. (2019). Spatial–Temporal Sensing and Utilization in Full Duplex Spectrum-Heterogeneous Cognitive Radio Networks for the Internet of Things. Sensors.

[3] Siddiqi M.A., Yu H., Joung J. (2019). 5G Ultra-Reliable Low-Latency Communication Implementation Challenges and Operational Issues with IoT Devices. Electronics.

[4] Zikria Y.B., Yu H., Afzal M.K., Rehmani M.H., Hahm O. (2018). Internet of Things (IoT): Operating System, Applications and Protocols Design, and Validation Techniques. Future Gener. Comput. Syst..

[5] Khalid W., Yu H., Noh S. (2020). Residual Energy Analysis in Cognitive Radios with Energy Harvesting UAV under Reliability and Secrecy Constraints. Sensors.

[6] Raghavan V., Li J. (2019). Evolution of Physical-Layer Communications Research in the Post-5G Era. IEEE Access.

[7] Yu H., Afzal M.K., Zikria Y.B., Rachedi A., Fitzek F.H.P. (2020). Tactile Internet: Technologies, test platforms, trials, and applications. Future Gener. Comput. Syst..

[8] Bariah L., Mohjazi L., Muhaidat S., Sofotasios P.C., Kurt G.K., Yanikomeroglu H., Dobre O.A. (2020). A Prospective Look: Key Enabling Technologies, Applications and Open Research Topics in 6G Networks. IEEE Access.

[9] Khalid W., Yu H. (2018). Sum Utilization of Spectrum with Spectrum Handoff and Imperfect Sensing in Interweave Multi-Channel Cognitive Radio Networks. Sustainability.

[10] Khalid W., Yu H. (2017). Optimal Sensing Performance for Cooperative and Non-Cooperative Cognitive Radio Networks. Int. J. Distrib. Sens. Netw..

[11] Zhao L., Liang T., An K. (2020). Performance Optimization of Hybrid Satellite-Terrestrial Relay Network Based on CR-NOMA. Sensors.

[12] Darsena D., Gelli G., Iudice I., Verde F. (2020). Separable MSE-Based Design of Two-Way Multiple-Relay Cooperative MIMO 5G Networks. Sensors.

[13] Rong Y. (2011). Joint Source and Relay Optimization for Two-Way MIMO Multi-Relay Networks. IEEE Commun. Lett..

[14] Khalid W., Yu H. (2021). Security Improvement With QoS Provisioning Using Service Priority and Power Allocation for NOMA-IoT Networks. IEEE Access.

[15] Do D.T., Le A.T., Kharel R., Silva A., Shattal M.A. (2020). Hybrid Satellite-Terrestrial Relay Network: Proposed Model and Application of Power Splitting Multiple Access. Sensors.

[16] Arshad J., Rehman A., Rehman A.U., Ullah R., Hwang S.O. (2020). Spectral Efficiency Augmentation in Uplink Massive MIMO Systems by Increasing Transmit Power and Uniform Linear Array Gain. Sensors.

[17] Khalid S., Abbas W.B., Kim H.S., Niaz M.T. (2020). Evolutionary Algorithm Based Capacity Maximization of 5G/B5G Hybrid Pre-Coding Systems. Sensors.

[18] Diyan M., Khan M., Nathali Silva B., Han K. (2020). Scheduling Sensor Duty Cycling Based on Event Detection Using Bi-Directional Long Short-Term Memory and Reinforcement Learning. Sensors.

[19] Kim T., Park S. (2020). Statistical Beamforming for Massive MIMO Systems with Distinct Spatial Correlations. Sensors.

[20] Khalid W., Yu H. Residual Energy Analysis with Physical-Layer Security for Energy-Constrained UAV Cognitive Radio Systems. In Proceedings of the 2020 International Conference on Electronics, Information, and Communication (ICEIC).

[21] Toan H.V., Hoang T.M., Duy T.T., Dung L.T. (2020). Outage Probability and Ergodic Capacity of a Two-User NOMA Relaying System with an Energy Harvesting Full-Duplex Relay and Its Interference at the Near User. Sensors.

[22] Kamal M.S., Kader M.F., Islam S.M.R., Yu H. (2020). Device-to-Device Aided Cooperative Relaying Scheme Exploiting Spatial Modulation: An Interference Free Strategy. Sensors.

[23] Mesleh R.Y., Haas H., Sinanovic S., Ahn C.W., Yun S. (2008). Spatial Modulation. IEEE Trans. Veh. Technol..

[24] Khan M., Seo J., Kim D. (2020). Towards Energy Efficient Home Automation: A Deep Learning Approach. Sensors.

[25] Jamshed M.A., Ur-Rehman M., Frnda J., Althuwayb A.A., Nauman A., Cengiz K. (2020). Dual Band and Dual Diversity Four-Element MIMO Dipole for 5G Handsets. Sensors.

[26] Bechta K., Kelner J.M., Ziółkowski C., Nowosielski L. (2020). Inter-Beam Co-Channel Downlink and Uplink Interference for 5G New Radio in mm-Wave Bands. Sensors.

[27] Khalid W., Yu H., Joung J. Physical Layer Security for Hybrid-ARQ Protocols with Massive Antennas. In Proceedings of the 2020 KICS Winter Conference.

[28] Verdecia-Peña R., Alonso J.I. (2021). A Two-Hop mmWave MIMO NR-Relay Nodes to Enhance the Average System Throughput and BER in Outdoor-to-Indoor Environments. Sensors.

[29] Chikha H.B., Almadhor A., Khalid W. (2021). Machine Learning for 5G MIMO Modulation Detection. Sensors.

[30] Imoize A.L., Adedeji O., Tandiya N., Shetty S. (2021). 6G Enabled Smart Infrastructure for Sustainable Society: Opportunities, Challenges, and Research Roadmap. Sensors.

